# Suppressing glucose uptake and acetic acid production increases membrane protein overexpression in *Escherichia coli*

**DOI:** 10.1186/1475-2859-10-35

**Published:** 2011-05-17

**Authors:** Emma Bäcklund, Marina Ignatushchenko, Gen Larsson

**Affiliations:** 1 Div of Bioprocess Technology, School of Biotechnology, Albanova University Center, Royal Institute of Technology, SE 106 91 Stockholm, Sweden; 2 Department of Medical Biochemistry and Biophysics, Karolinska Institute, SE 171 77 Stockholm, Sweden

## Abstract

**Background:**

The production of integral membrane spanning proteins (IMP's) constitutes a bottleneck in pharmaceutical development. It was long considered that the state-of-the-art was to produce the proteins as inclusion bodies using a powerful induction system. However, the quality of the protein was compromised and the production of a soluble protein that is incorporated into the membrane from which it is extracted is now considered to be a better method. Earlier research has indicated that a slower rate of protein synthesis might overcome the tendency to form inclusion bodies. We here suggest the use of a set of *E. coli *mutants characterized by a slower rate of growth and protein synthesis as a tool for increasing the amount of soluble protein in high- throughput protein production processes.

**Results:**

A set of five IMP's was chosen which were expressed in three mutants and the corresponding WT cell (control). The mutations led to three different substrate uptake rates, two of which were considerably slower than that of the wild type. Using the mutants, we were able to express three out of the five membrane proteins. Most successful was the mutant growing at 50% of the wild type growth rate. A further effect of a low growth rate is a low acetic acid formation, and we believe that this is a possible reason for the better production. This hypothesis was further supported by expression from the BL21(DE3) strain, using the same plasmid. This strain grows at a high growth rate but nevertheless yields only small amounts of acetic acid. This strain was also able to express three out of the five IMP's, although at lower quantities.

**Conclusions:**

The use of mutants that reduce the specific substrate uptake rate seems to be a versatile tool for overcoming some of the difficulties in the production of integral membrane spanning proteins. A set of strains with mutations in the glucose uptake system and with a lower acetic acid formation were able to produce three out of five membrane proteins that it was not possible to produce with the corresponding wild type.

## Background

The production of recombinant proteins is generally considered to be a burden on the host cell. In severe cases, protein expression cannot be pursued at all or leads to the accumulation of degraded protein or of protein which is precipitated in the form of inclusion bodies. At the same time, expression may be accompanied by a high degree of cell death in the population that reduces the total yield and productivity. Except for rare cases, it is difficult to pinpoint the exact mechanism behind this malfunction. Each protein is unique and the steps in the synthesis of an active protein are many.

A common way to overcome expression difficulties is to lower the temperature, which is a parameter that can be used regardless of expression system and protein. The reason why this is efficient in a particular case is however difficult to identify since the temperature affects a large number of cellular mechanisms as well as all the equilibrium constants. A temperature decrease will thus have an impact on both positive and negative reactions controlling the specific protein production, and this explains why this strategy is often not successful. The reduction in temperature however always leads to a slower growth rate and thus a slower rate of protein synthesis in the cell. Some researchers have suggested that a lower synthesis rate will allow the cell to overcome important bottlenecks such as folding and the rate of translocation between cell compartments. Especially in membrane protein production, it has been clearly shown that when a strong induction system like the BL21(DE3) strain is used, it is indeed the high and immediate rate of protein-specific mRNA accumulation, resulting from high level T7 RNA polymerase production, that has detrimental effects on the host cell and thus lowers the product yield [1-3].

We have earlier shown that a reduction in the growth rate, in our case caused not by a temperature reduction but by a restricted feed of the carbon source, has a large impact on recombinant protein production [4-6]. A lower growth rate was shown to result in a lower cell-specific rate of production of the recombinant protein although the specific mRNA level, i.e. the transcription rate, was the same. The lower rate of protein synthesis here was probably due to a lower translation rate since the lower substrate uptake rate resulted in a smaller amount of ribosomes. The total protein production was not however impaired since a longer cultivation time led to the same amount of protein accumulation. The great benefits of the system were less proteolytic degradation of the protein and less inclusion body formation. The relatively slower synthesis rate of the recombinant protein is thus important for the quality of the protein product, and it seems to be particularly important in the production of membrane proteins which are preferably produced in a soluble membrane fraction [7].

During the development of new pharmaceutical drugs, one of the goals is to rapidly deliver small soluble quantities of a large number of proteins and protein variants for further studies of their structure and function in relation to disease control. To avoid bottlenecks, the most favorable conditions are chosen with respect to expression system and medium. In order to screen a number of different proteins, production takes place in a multiparallel unsupervised format (HTP, High Throughput Protein production) which leads to uncontrolled process conditions with respect to important parameters such as pH, acetic acid production and oxygenation, and therefore to low cell densities and low product concentrations. No variation in the production scheme can be allowed and all clones are induced simultaneously and are harvested at the same time, which means that the cultivation time for all the cells is the same.

The development of a commercial protein production process, on the other hand, allows the measurement and control of a number of parameters, resulting in a range of actions that can be taken to increase the productivity and to keep important parameters constant. The process is carefully sampled and optimized during the process development. The most important control technique used is the fed-batch technique, which allows the accumulation of high cell densities but also the metabolic control of acetic acid production. This is achieved by operator control of the supply of the glucose which is fed to the cultivation, in order to restrict the glucose uptake by the individual cell.

In order to take advantage of the important process control strategies of the commercial production process during HTP, we have used a set of *E. coli *mutants which are deficient in their glucose uptake due to deletions in the phosphoenolpyruvate:phosphotransferase system (PTS). Since these cells have a reduced glucose uptake, they have on a cellular level all the advantages generally associated with the fed-batch concept [8]. The restricted rates of growth and substrate uptake of these cells were shown to affect not only the growth rate but also the formation of overflow byproducts like acetic acid. Earlier studies have shown that this acid not only limits the cell density but also has a major impact on the growth rate and on the production of human growth hormone in *E. coli *[9].

The goal of the present work was to understand whether the mutants in the glucose uptake system could be used under the conditions which characterise the small-scale multi-parallel production format and thus lead to greater success in soluble protein accumulation. The targets of the investigation were integral membrane spanning proteins (IMP's). We also aimed to ascertain whether it is the substrate uptake rate or the acetic acid formation that has the greater influence on the soluble IMP production.

## Materials and methods

### Cloning

A blunt-ended GW-cassette (from Invitrogen's Gateway Conversion kit) was inserted into the *Sma*I-digested pBluescript intermediate construct yielding pBS-GW. A clone with the correct orientation of the GW-cassette in frame A was chosen by digestion with *Eco*I. pBADHisA (Invitrogen) was digested with *Nhe*I and *Hind*III. pBS-GW was digested with *Spe*I and *Hind*III and was ligated into the precut pBADHisA, yielding BADHisA-frame A GW.

This construct was transformed into DB3.1 cells (Z-competent, in-house production). Transformants were grown on LB agar plates containing 34 μg/ml chloramphenicol. Colonies were selected and grown in liquid cultures for subsequent plasmid preparation (QIAprep Spin Miniprep Kit (Qiagen)). The plasmids were test digested with *Nco*I. A clone with the correct size of the digested fragment was sent for sequencing (Eurofinsdna, Germany) to verify the correct insertion of the GW-cassette.

Vectors with protein targets EM03, EM09, EM16, EM20, EM29 [7] were used as entry clones. Site-specific recombination catalysed by the LR clonase enzyme mix (Invitrogen) was used in order to subclone the entry clones into the destination vector pBADHisA-frame A GW. The identities of the constructs were verified by sequencing (Eurofinsdna, Germany).

The resulting expression vectors, all expressing the proteins from the arabinose promoter, were transformed into *E. coli *BL21(DE3) (derived from the original Novagen stock), AF1000 (MC4100 *relA^+^*), PPA668 (*ptsM*), PPA652 (*ptsG*) and PPA689 (*ptsG, ptsM*) [8].

### Cultivation medium

A minimal salt media was used which consisted of (per litre): 7 g (NH_4_)_2_SO_4_; 1.6 g KH_2_PO_4_; 6.6 g Na_2_HPO_4_*2H_2_O and 0.5 g (NH_4_)_2_-H-Citrate. The medium was supplemented with 1 ml/l trace element solution [8] and1 ml/l 1 M MgSO_4 _and 10 g/l glucose. 100 μg/ml carbenicilin was added to cells containing plasmids.

### Small-scale protein overexpression

200 μl of overnight culture was used to inoculate 1.8 ml of medium. The cells were grown in 24-well plates at 37°C at 1350 rpm. Induction was performed by the addition of 0.2% arabinose when the cells had reached an OD_600 _of approximately 0.5. 1 ml of the cultures was harvested three hours after the induction. The pellets were stored at -20°C.

### Small-scale protein purification

Small-scale IMAC with Ni^2+^-resin was used for purification of the proteins in 96-well format. 25 μl Ni-NTA (Invitrogen) was added to a 0.65 μm filter plate (Millipore) that was centrifuged for 1 min at 100 g. The plate was washed with 100 μl mQ-H_2_O and equilibrated with 200 μl wash buffer (20 mM Tris-HCl, 300 mM NaCl, 40 mM Imidazole, 0.1% FC12 (Anatrace) and 0,5 mM TCEP).

The pellets from the small-scale cultivation were thawed and resuspended in 100 μl lysis buffer (20 mM Tris pH 8, 100 mM NaCl, 1 mg/ml lysozyme (Sigma), 10 U/ml benzonase (Novagen), 1% FC12 and one tablet Complete Protease Inhibitor EDTA-free (Roche) per 22 ml buffer). The suspension was incubated at 1000 rpm at 4°C for 30 min and centrifuged for 30 min at 3000 g at 4°C.

The Ni-plate was prepared by centrifugation for 1 min at 100 g. The clarified cell lysate was added to the Ni-plate that was then incubated for 15 min at 4°C at 600 rpm and centrifuged for 1 min at 100 g. The resin was washed three times with 200 μl wash buffer and eluted by addition of 25 μl elution buffer (20 mM Tris-HCl pH 8, 300 mM NaCl, 500 mM Imidazole, 0,5 mM TCEP and 0.1% FC-12) followed by centrifugation at 100 g for 1 min at 4°C.

### Dot blot analysis

1.5 μl of the eluate was applied to a nitrocellulose membrane and allowed to dry. The membrane was incubated overnight at 4°C in a blocking solution consisting of 1% BSA in TBS-T pH 7.5. The membranes were washed three times for 10 min with TBS-T and incubated for one hour with His-probe-HRP (Pierce) diluted 1:5000 in TBS-T. The membranes were washed three times for 10 min with TBS-T and developed with Super Signal^® ^West Pico (Pierce) according to the manufacturer's recommendations. The signals were detected with the FluorS-MultiImager (BioRad).

### Medium-scale protein overexpression

Target EM03 was expressed in medium-scale liquid cultures (400 ml). 360 ml medium was inoculated with 40 ml of the overnight culture. The cells were grown at 37°C at 200 rpm until they reached an OD_600 _of approximately one unity when induction with 0.2% arabinose took place. OD_600 _was measured and cells from a 100 ml suspension were harvested by centrifugation at 5000 g for 10 min at 4°C three hours after the induction. The pellets were stored at -80°C.

### Membrane protein extraction

The frozen pellets were thawed. 15 ml of lysis buffer (20 mM Na-P, 300 mM NaCl, 0.5 mM DTT, 5 U/mL DNase, 1 mg/ml lysozyme, 30 mM Imidazole and one tablet Complete Protease Inhibitor EDTA-free per 100 ml buffer) was added to each pellet. The suspension was agitated at 1000 rpm at 4°C for 40 min. The samples were sonicated and centrifuged at 10000 g for 10 min at 4°C. 7.5 ml of the supernatant was centrifuged at 35000 g for one hour in order to harvest the membranes. The membrane pellet was resuspended in 3 ml solubilisation buffer (20 mM Na-P, 300 mM NaCl, 30 mM Imidazole pH 7.5, 0.5 mM DTT and one tablet Complete Protease Inhibitor EDTA-free per 100 ml) per 200 OD_600 _units of former cells.

The membranes were resuspended by shaking and homogenised using a glass homogenizer. A small aliquot was taken for SDS-PAGE analysis. The samples were mixed with NuPAGE^® ^Sample Reducing Agent and NuPAGE^® ^LDS Sample buffer (Invitrogen). 15 μl of the samples were run on a NuPAGE^® ^4-12% Bis-Tris gel using NuPAGE^® ^MES buffer system (Invitrogen). SeeBlue^® ^Plus2 (Invitrogen) was used as marker. The gels were stained with Coomassie brilliant blue to visualize the protein bands. The remaining samples were stored at -80°C.

### Medium-scale protein purification

FC-12 was added to the thawed samples to a final concentration of 1% following incubation during shaking at 1000 rpm at 4°C and centrifugation at 30000 g for 30 min at 4°C. 250 μl Ni-NTA agarose resin was prepared by washing with 5 ml water and 2 ml elution buffer (20 mM Na-P, 150 mM NaCl, 400 mM Imidazole, pH 7.5, 0.5 mM DTT, 0.1% FC12) and equilibrated with 2 ml wash buffer 1 (20 mM Na-P, 300 mM NaCl, 30 mM Imidazole, pH 7.5, 0.5 mM DTT, 0,1% FC12). The samples were added to the resins and incubated for 10 min at 4°C, followed by the addition of wash buffer 1 (2 × 2 ml) and wash buffer 2 (2 × 2 ml) (20 mM Na-P, 300 mM NaCl, 40 mM Imidazole, pH 7.5, 0.5 mM DTT, 0.1% FC-12). Bound proteins were eluted with 1500 μl elution buffer. The samples were concentrated to a volume of 250 μl using a 30 kDa cut-off filter. A small aliquot of each sample was analyzed by SDS-PAGE as described above. 15 μl of each sample was loaded to lane 2, 3, 5 and 6 and 7 μl to lane 4.

### Analytical gel filtration

15 μl of the samples were loaded in duplicates on a Superdex™ 200 5/150 GL column (GE Healthcare). The buffer was Hepes pH 7.5. The yields were calculated by measuring the absorbance at 280 nm. The chromatogram shows values for the same amounts of OD_600 _units of cells, normalized so that the top peak absorbance was 1.

The areas beneath the curves in the chromatograms were calculated in order to compare the amounts of protein produced in the different strains. The diagram shows peak areas for the same amounts of OD_600 _units of cells, normalized so that the top peak area was 1.

### Acetate production

The cells were grown in 500 ml medium at 37°C at 180 rpm, in order to determine the amount of acetic acid produced per OD_600 _unit of cells. A 6 ml sample of cell suspension was taken every hour. 1 ml was used to determine the OD_600 _and 5 ml was centrifuged for 10 min at 4500 rpm at 4°C. The supernatant was sterile filtered (Sartorius, 0.2 μm) and the acetate concentration was determined using a commercial enzymatic kit (Boehringer-Mannheim no. 148261). Each sample was tested four times and the mean value was calculated.

## Results

The aim of this study was to understand whether a reduction in the growth rate/substrate uptake rate would lead to a greater success in the production of soluble integral membrane spanning proteins in *E. coli*. For this purpose we used a set of strains that was constructed [10] to carry deletions in the phosphoenolpyruvate carbohydrate:phosphotransferase system (PTS), responsible for the uptake of various sugars in *E. coli *[11]. These strains and the corresponding wild type (AF1000), which are K-derivatives, are listed in Table 1. The mutants are characterized by their inability to take up glucose through the deleted permeases: (i) a defective enzyme IIAB^Man^, which unspecifically controls the uptake of mannose but also allows glucose passage (*ptsM*), (ii) a defective enzyme IIBC^Glc ^(*ptsG*), specific for glucose uptake, and (iii) the double mutant (*ptsG,ptsM*), in which glucose is taken up by symport. The commercial strain BL21(DE3), which is a B-derivative, was also included since this strain is often used for the production of IMP's [1,7,12,13] The production was controlled by the same vector as for the AF1000 strains.

**Table 1:** Strain characterization

Strain	Specific growth rate	Mutation
	(h^-1^)	
AF1000	0,05	-
PPA668	0,78	ptsM
PPA652	0,38	ptsG
PPA689	0,25	ptsG, ptsM
BL21(DE3)	0,76	-

Strains used in the study and their corresponding specific growth rates in minimal salt medium supplemented with glucose at 37°C.

The effect of the mutations on growth rate is shown in Table 1. It is clear that the removal of the glucose permease (*ptsG*) effectively reduces the rate of growth at high glucose concentrations. It should be noted that a minimal salts medium was used with glucose as carbon source and that no complex components were added to the medium, in order to avoid the interference of e.g. a lack of specific amino acids on the protein production.

Five *E. coli *membrane spanning integral proteins (IMP's) with human homologues were selected for the study and were produced with N-terminal His_6 _tags to facilitate purification. The proteins and their characteristics are listed in Table 2. Some of these proteins were considered difficult to express in earlier studies [7] using the C41(DE3) strain, i.e. the BL21(DE3) mutant first described by Miroux and Walker [1].

**Table 2:** Membrane protein characteristics

Target	NCBI Reference Sequence	Predicted Function
EM03	ZP_03069574.1	Ammonium transporter
EM09	NP_418444.1	Predicted transporter
EM16	ABV06797.1	Chloride transporter, chloride channel (CIC) family
EM20	NP_418166.1	Multidrug efflux system protein
EM29	NP_756065.1	Intramembrane serine protease GlpG

The integral membrane proteins used in the study.

**Figure 1:**
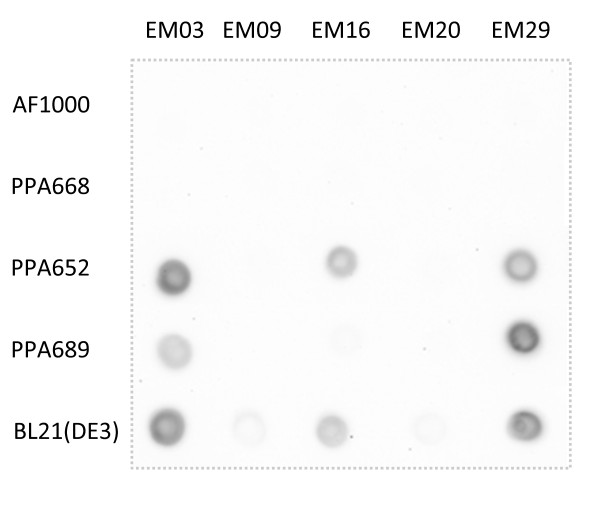
**Expression of membrane proteins**. Dot blot of purified His-tag proteins overexpressed in the following strains: AF1000, PPA668, PPA652, PPA689 and BL21(DE3). See Table 1 for the strain characteristics. The cells were grown on microtitre plates and were induced with 0.2% arabinose at 37°C.

The IMP's were cloned and induced from the arabinose promoter in all strains and grown on microtitre plates. A dot blot was used to show the expression of the targets in the different strains (Figure 1). The mutants carrying the glucose permease mutation were able to produce three out of the five proteins, as was the BL21(DE3) strain. There are no obvious reasons for the difficulty in producing the two remaining proteins, at least not with respect to the amount of α-helix membrane spanning units, which varied in a random fashion from six to twelve. The cells were induced at an OD_600 _of approximately one and were all harvested after the same time of expression (three hours). The amounts of cells at the time of harvest varied since the strains have different growth rates. A quantitative comparison of the expression levels in the different strains is not therefore possible from this dot blot.

**Figure 2:**
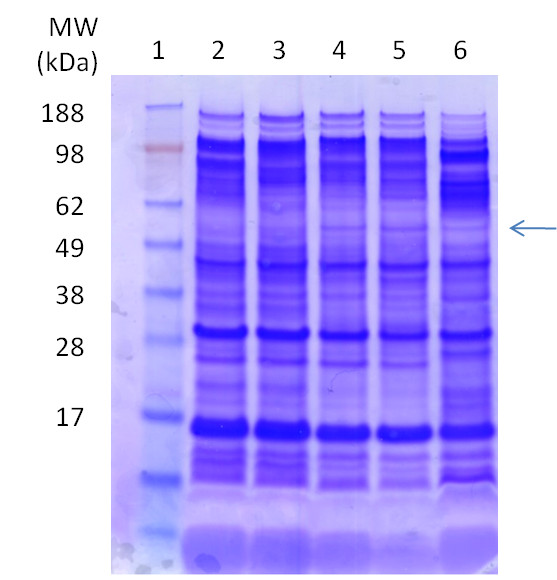
**Analysis of the membrane protein fraction**. SDS-PAGE analysis containing membrane fractions from medium-scale shake flask cultivations. The target EM03 is indicated by an arrow. The lanes were loaded in the following way: 1 marker, 2 AF1000, 3 PPA668, 4 PPA652, 5 PPA689, 6 BL21(DE3).

**Figure 3:**
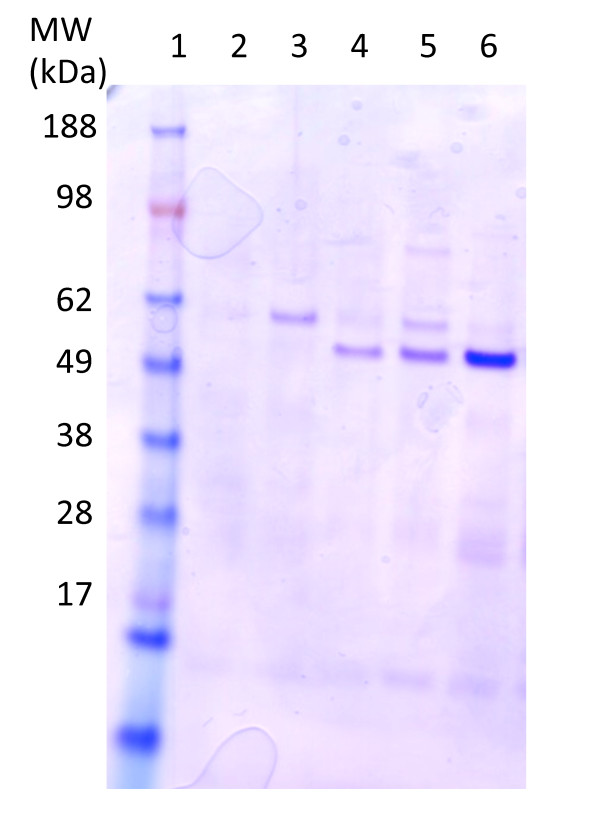
**Verification of expression of the target protein**. SDS-PAGE analysis of IMAC-purified target EM03. The lanes were loaded in the following way: 1 marker, 2 AF1000, 3 PPA668, 4 PPA652, 5 PPA689, 6 BL21(DE3).

In order to be able to compare the relative amounts of IMP's and to assess the degree of homogeneity of the target proteins, we scaled up the production of the EM03 protein into shake flasks. The composition of the membrane protein isolation fraction is shown by SDS-PAGE in Figure 2. The EM03 48 kDa protein was produced only by the glucose permease mutants and the BL21(DE3) strain, which supports the dot blot results. The samples were subjected to IMAC resin in order to purify the His_6_-tagged product. The expression of the target protein was verified by Comassie-stained SDS-PAGE (Figure 3).

**Figure 4:**
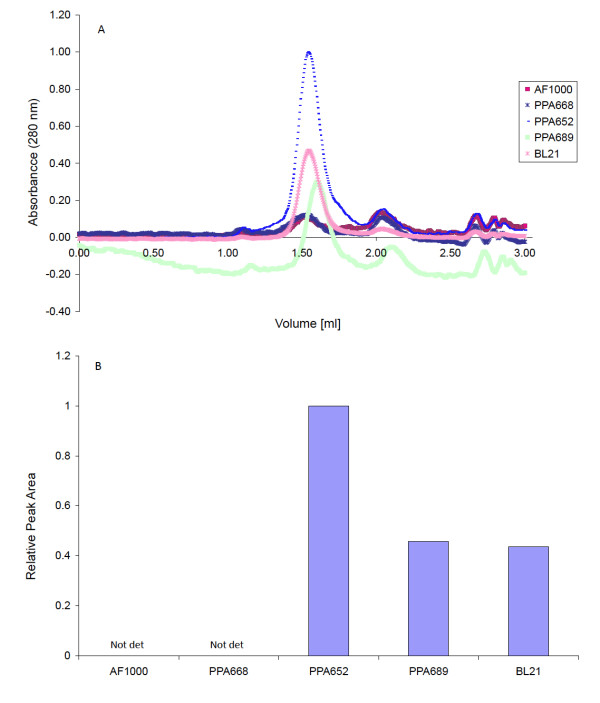
**Purification and quantification of the selected membrane protein target**. (A) Analytical gel filtration from medium-scale shake flask cultivations using the target protein, EM03. The chromatogram shows values for an equal OD_600 _-value of cells in the different samples, normalized with respect to the highest top peak absorbance set equal to one. (B) Estimated peak areas of analytical gel filtration chromatograms from the production of the target EM03. The values show calculated peak areas for an equal OD_600 _-value of cells in the different samples, normalized with respect to the largest top peak area set equal to one.

The samples were further subjected to analytical gel filtration to allow a quantitative comparison of the expression levels in the different strains. The gel filtration chromatogram is shown in Figure 4A where values for the same amounts of cells are shown normalized so that the top peak (maximum) value equals 1. The chromatogram shows that EM03 could be purified as a non-aggregate.

The amounts of EM03 that were produced in the different strains was calculated from the areas under the curves and the results are plotted in Figure 4B. The comparison shows that the glucose permease mutant produced approximately twice as much protein as the double mutant and BL21(DE3). The wild type and the mannose permease mutant, which both take up glucose at the same high rate, produced very small quantities of EM03, so that it was not possible to determine the areas in a statistically relevant fashion.

Since the BL21(DE3) strain was qualitatively as good a producer as the glucose permease mutants, in spite of its high specific growth rate, additional experiments were performed to clarify this issue. Since the lower substrate uptake in the mutants also leads to a lower production of acetic acid, the acetic acid production was further studied in all the strains. It was measured in shake cultivations and the yield of acid per cell is shown in Table 3. It is here notable that the BL21(DE3) strain had a comparatively low production of the acid at high glucose concentrations, although the growth rate was as high as in the wild type K-12 strain AF1000. The glucose permease mutants, on the other hand, show almost no overflow metabolism, although the glucose concentration is 10 g/L.

**Table 3:** Acetic acid production

Strain	HAc
	(mg/l) per OD_600_-unit
AF1000	158
PPA668	147
PPA652	3
PPA689	2
BL21(DE3)	58

Cell-specific acetic acid production of the strains used in the study.

## Discussion

*E. coli *BL21(DE3) seems to be the dominant choice for the production of membrane proteins for the purpose of solving their structures and the characterization of their functions [12]. Membrane proteins are in some cases produced by this strain, but the product usually appears as inclusion bodies [1], and this is generally undesirable due to downstream problems [12]. It is also clear that relatively few membrane protein structures have today been solved and furthermore that until 2005, more than 70% of the solved structures were of non-eukaryotic origin [12]. The lack of good protein production methods is thought to be one of the dominant factors behind the low success [13].

Miroux and Walker have shown that the production of eukaryotic membrane proteins with the BL21(DE3)-system is generally very toxic to the cells and that most of them did not even survive induction [1]. They thus developed the BL21(DE3) mutant strains, C41(DE3) and C43(DE3), the "Walker strains", which seems to be much better suited for production. Through subsequent genomic and proteomic studies [3], it was shown that the original P*_lacUV5_*promoter of these strains, which is generally stronger than the original P*_lac _*variant, had reverted to the original wild type sequence. It was clearly shown that the lower strength of the *lac *promoter led to a lower transcription rate of the T7 RNA polymerase, and that this in turn, had a positive effect on the cellular viability leading to higher total production levels. The mechanism by which the viability is restored has not yet been revealed. Wagner and coworkers suggested that it was due to the lower rate of transcription of the membrane protein, due to a decreased T7RNAP synthesis, and used this to modulate these levels using a tunable T7Lys expression system [3]. This resulted in increased cell viability and in several cases also to an increase in the total production. In some cases, however, the use of C41(DE3) and C43(DE3) gave still better results. The tuning of T7RNAP levels was already suggested by Miroux and Walker [1], but with the comment that it might not be altogether successful for the difficult eukaryotic membrane proteins that still remain to be successfully produced.

In this paper, we have described a different approach to membrane protein production by reducing the substrate uptake rate, and thus indirectly the protein synthesis rate, using the PTS-mutants. The production is introduced on low copy number plasmids with tunable promoter properties. The advantage, besides a lower synthesis rate, is that it avoids the ATP-costly transcription of specific messengers, which we believe to be an additional reason for the reduction in cell viability upon induction. In many cases in the literature it has been indicated that the cell after induction is lacking energy and will seek alternative sources for the supply of both carbon and energy, which can lead to e.g. ribosome degradation since these are by far the largest cellular resources available [5,14]. Energy-costly processes should thus be avoided as far as possible to save the cell from internal degradation.

Another large drawback of production systems involving elements of periplasmic or outer membrane transport is the possible saturation of the inner membrane secretory system (Sec), and this seemed to be another consequence of high and rapid membrane protein production in the BL21(DE3), pET-vector based system [2]. The accumulation of precursors of periplasmic and outer membrane proteins in the cytosol is of course also a waste of building blocks and energy. In the same study it was also shown that overexpression leads to the induction of the acetate-phosphotransacetylate pathway probably because of the need for additional energy which, under these particular circumstances, might not be possible to obtain by increased oxidative phosphorylation.

The glucose permease mutants and the BL21(DE3) strain, all induced by arabinose, are described in this paper as being equally effective in protein expression, as we were able to express three out of five proteins with all of them. However, the yield of protein per cell for the PPA652 mutant was twice as high as for the other producing systems. The glucose permease mutants grow more slowly than the BL21(DE3) cells and therefore need approximately twice the time (approx 7 h) to reach the same cell density. The mutant cells can however grow to a higher cell density since they produce no acetic acid, and this may lead to an even further increase in the total production level. This ability has earlier been shown when mutants were grown in a fermenter to an OD_600 _of approximately 100 [8]. The limit is set by the oxygen-transfer capacity, which in the fermenter is characterized by a K_L_a of approx 800 h^-1 ^compared to microtitre plates with a K_L_a of approximately 130 - 188 h^-1 ^[15,16]. Theoretically, this comparison should allow an OD_600 _of approximately 20 on the best plates using the mutants when no acetic acid is formed, which is about 5 times higher than the density reached today.

The question of whether it is the low growth rate or the lack of acetic acid production that is the reason for the better production has not been totally clarified. The data suggest the latter, since BL21(DE3), in spite of its high growth rate, shows similar production results to our glucose permease mutants, and the reason could then be the low acetic acid production of BL21(DE3) at high glucose concentrations in minimal medium. The reason why this B strain is not subject to overflow metabolism in the same way as the K-12 strains has, to our knowledge, not been explained in detail. It has however been suggested that B-strains have a more active glyoxylate shunt than K-12 strains [17], which results in the direction of pyruvate into other metabolic functions and in the reduction of acetic acid formation.

The comparative data presented in Figure 4 and Table 3 also suggest that the lower the acetic acid production the higher is the protein production. This is not however the only factor that is important, since it seems that a too low glucose uptake, maybe only leading to maintenance levels of glucose in the double mutant, also reduces production. This again suggests the importance of a high-energy status of the cell.

## Conclusions

The use of PTS mutants that slow down the specific growth rate seems to be a versatile tool to overcome some of the difficulties in the production of integral membrane spanning proteins. A set of strains with mutations in the glucose PTS-uptake system exhibiting a lower acetic acid production, were able to produce three out of five membrane proteins, which was impossible with the corresponding wild type. Not only were these proteins expressed but also in relatively larger quantities than with the commonly used BL21(DE3) strain using the same induction system. This means that this methodology can be used for other membrane proteins, as well as for difficult-to-express proteins that are desired to be soluble in the cytoplasm.

## Competing interests

The authors declare that they have no competing interests.

## Authors' contributions

EB has made contributions to the design of the experiments, the acquisition of data, the analysis and interpretation of data and has contributed to the writing of the manuscript. GL has made contributions towards the conception and design of the project and to the writing of the manuscript. MI has made substantial contributions towards the choice of analytical techniques, the analysis and the data interpretation and has revised the manuscript. All the authors have given their final approval of the version to be published.
